# Effect modification of consecutive high concentration days on the association between fine particulate matter and mortality: a multi-city study in Korea

**DOI:** 10.4178/epih.e2022052

**Published:** 2022-06-09

**Authors:** Hyungryul Lim, Sanghyuk Bae, Jonghyuk Choi, Kyung-Hwa Choi, Hyun-Joo Bae, Soontae Kim, Mina Ha, Ho-Jang Kwon

**Affiliations:** 1 Department of Preventive Medicine, Dankook University College of Medicine, Cheonan, Korea; 2 Department of Preventive Medicine, College of Medicine, The Catholic University of Korea, Seoul, Korea; 3 Korea Environment Institute, Sejong, Korea; 4 Department of Environmental and Safety Engineering, Ajou University, Suwon, Korea

**Keywords:** Air pollution, Particulate matter, Epidemiology, Mortality, Time-series analysis

## Abstract

**OBJECTIVES:**

Although there is substantial evidence for the short-term effect of fine particulate matter (PM_2.5_) on daily mortality, few epidemiological studies have explored the effect of prolonged continuous exposure to high concentrations of PM_2.5_. This study investigated how the magnitude of the mortality effect of PM_2.5_ exposure is modified by persistent exposure to high PM_2.5_ concentrations.

**METHODS:**

We analyzed data on the daily mortality count, simulated daily PM_2.5_ level, mean daily temperature, and relative humidity level from 7 metropolitan cities from 2006 to 2019. Generalized additive models (GAMs) with quasi-Poisson distribution and random-effects meta-analyses were used to pool city-specific effects. To investigate the effect modification of continuous exposure to prolonged high concentrations, we applied categorical consecutive-day variables to the GAMs as effect modification terms for PM_2.5_.

**RESULTS:**

The mortality risk increased by 0.33% (95% confidence interval [CI], 0.16 to 0.50), 0.47% (95% CI, -0.09 to 1.04), and 0.26% (95% CI, -0.08 to 0.60) for all-cause, respiratory, and cardiovascular diseases, respectively, with a 10 μg/m^3^ increase in PM_2.5_ concentration. The risk of all-cause mortality per 10 μg/m^3^ increase in PM_2.5_ on the first and fourth consecutive days significantly increased by 0.63% (95% CI, 0.20 to 1.06) and 0.36% (95% CI, 0.01 to 0.70), respectively.

**CONCLUSIONS:**

We found increased risks of all-cause, respiratory, and cardiovascular mortality related to daily PM_2.5_ exposure on the day when exposure to high PM_2.5_ concentrations began and when exposure persisted for more than 4 days with concentrations of ≥35 μg/m^3^. Persistently high PM_2.5_ exposure had a stronger effect on seniors.

## GRAPHICAL ABSTRACT

[Fig f3-epih-44-e2022052]

## INTRODUCTION

The background level of fine particulate matter (PM_2.5_) concentration is much higher in Korea than in North America and Western Europe [1]. According to the Korean Ministry of Environment, the average annual concentration of PM_2.5_ in all regions of Korea in 2019 was 23 μg/m^3^. In 2011, the Korean government established new air quality guidelines for PM_2.5_, which were strengthened in March 2018 to advise a daily average of 35 μg/m^3^ instead of 50 μg/m^3^ and an annual average of 15 μg/m^3^ instead of 25 μg/m^3^ [2]. Consequently, the public has been notified on days when this standard is exceeded. Moreover, when the hourly average concentration exceeds 75 μg/m^3^ or 150 μg/m^3^ for more than 2 hours, an advisory or alert is issued regionally, which has increased public concern about the health effects of PM_2.5_.

In epidemiological studies on the health effects of air pollution, exposure is generally classified as both short-term and long-term. The former refers to exposure over a period ranging from a few hours to a month (mostly within the same day to 1 week), and the latter refers to exposure over a period of more than 1 month to several years [3].

Despite its heterogeneity across different cities worldwide, several multi-city time-series studies of the short-term effects of ambient PM_2.5_ exposure on daily mortality have consistently observed statistically significant effects [4-7], and evidence for causality has been reported in recent studies [8,9]. Although the background PM_2.5_ concentration in Korea has shown a decreasing trend since these levels started to be measured in 2015 [10], prolonged exposure to a high concentration of PM_2.5_ has frequently been observed. The average duration of exposure to a PM_2.5_ concentration of 35 μg/m^3^ or above in the first quarter of the year in Korea reportedly increased from 16.2 hours in 2015 to 26.5 hours in 2018, and the average 1-hour concentration also increased slightly from 95.4 μg/m^3^ in 2015 to 102 μg/m^3^ in 2018 [11].

Thus, we reviewed the existing evidence on whether the mortality effect of short-term exposure to high concentrations of PM_2.5_ differs from that of short-term exposure to non-high concentrations of PM_2.5_, and, if so, whether the effect differed according to the length of exposure. However, relatively few observational studies have addressed the durational effects of continuous exposure to prolonged high concentrations of particulate matter (PM). Two existing previous studies found additional positive durational effects on daily mortality over several days [12,13].

We aimed to investigate the short-term effect of ambient PM_2.5_ on daily mortality in Korea and assess the effect of prolonged exposure to a high concentration of PM_2.5_ by examining whether the effect sizes were modified based on the duration of exposure.

## MATERIALS AND METHODS

### Study area and population

The study areas were Seoul, the capital city of Korea, and 6 other metropolitan areas: Busan, Daegu, Incheon, Gwangju, Daejeon, and Ulsan (Supplementary Material 1). The study population from these 7 areas was approximately 22.6 million in 2019, covering approximately 43.7% of the nation’s total population. The study was conducted from January 1, 2006, to December 31, 2019, for a total of 5,113 days.

### Mortality data

We obtained cause of death statistics data from the MicroData Integrated Service (https://mdis.kostat.go.kr/) of Statistics Korea, which publishes open-source mortality data available to the public for research purposes. We included daily non-accidental all-cause deaths (International Classification of Diseases, 10th edition [ICD-10], codes A00 to R99), deaths from respiratory disease (ICD-10 codes J00 to J98), and deaths from cardiovascular disease (I00 to I99) in 7 major cities from January 1, 2006, to December 31, 2019.

### Fine particulate matter mass concentration data

In Korea, ambient PM_2.5_ levels have been measured through the national air pollution monitoring network since 2015. Therefore, we used simulated data that considered weather conditions, anthropogenic and biogenic emissions, and chemical transport to estimate PM_2.5_ concentrations extending back to 2006 [14,15], as has been done in several recent epidemiological studies of air pollution in Korea [16,17].

We used the Community Multiscale Air Quality (CMAQ) system, version 4.7.1, with the AERO5 aerosol module and Statewide Air Pollution Research Center 99. Weather simulations were performed using the Weather Research and Forecasting model, version 3.3.1, with the National Center for Environmental Protection final data as the initial field. The Meteorology Interface Processor version 3.6 was used to prepare the CMAQ-ready meteorological inputs. The Clean Air Policy Support System 2010, which is the Korean national emissions inventory, was processed through the Sparse Matrix Operator Kernel Emission, version 3.1, to estimate anthropogenic emissions, and biogenic emissions were estimated using the Model of Emissions of Gases and Aerosols from Nature. We applied a 27-km (covering Northeast Asia, including China, Japan, and Korea) and a 9-km modeling domain. The 27-km modeling domain simulation values were applied to the boundary condition of the 9-km modeling domain. The CMAQ system simulated gridded hourly PM_2.5_ concentrations from the 9-km modeling domain for each of the 7 cities, which were resampled and averaged to allocate city-specific concentrations of PM_2.5_. Subsequently, we calculated the daily average PM_2.5_ concentration for each of the cities from January 1, 2006, to December 31, 2019.

### Meteorological data for covariates

We collected daily average temperature and relative humidity data (1 measurement point per city) for 7 major cities from January 1, 2006 to December 31, 2019, from an open-source dataset published by the National Climate Data Center of the Korea Meteorological Administration (https://data.kma.go.kr/).

### Statistical analysis

We first evaluated the association between daily PM_2.5_ concentration and daily non-accidental all-cause, respiratory disease, and cardiovascular disease mortality using time-series analyses as basic procedures. A generalized additive model (GAM) with a quasi-Poisson distribution was used as a statistical model, and the commonly applied model equation was as follows:


(1) $$\log[(E(Y_{t,c})]=\beta_{0,l,c}\;+\;\beta_{PM2.5,l,c}*PM_{2.5\;t,l,c}\;+\;s(temp_{t,c},\;df\;=\;6)\;+s(rh_{t,c},\;df\;=\;3)\;+s(time_{t},\;df\;=\;7\;*\;number\;of\;years)\;+\;\eta \;*\;factor\;(dow_{t})$$

Where *E*(*Y_t,c_*) is the expected death count on day *t* in city *c*; *PM_2.5, t, l, c_* is the average 24-hour mean PM_2.5_ concentration (μg/m^3^) on day *t* applied lag *l* (0 to 6 days before, and the 2-day to 7-day moving average, which means the average over the current and previous day and the current day to before the sixth day, respectively) in city *c*; *β_PM 2.5, l, c_* is the corresponding coefficient; *temp_t, c_* is the average 24-hour mean temperature (°C) on day *t* and day *t*-1 in city *c*; *rh_t, c_* is the mean relative humidity (%) on day *t* in city *c*, *time_t_* is a continuous variable-processed of day *t* to adjust the long-term trend, and *dow_t_* is a categorical day-of-week and holiday variable of day *t*, which has 4 levels (weekday, Sunday and holiday, the day after Sunday and holiday, and Saturday) [14], where *s* represents the smooth spline function, *df* represents the degrees of freedom applied to each function, and η represents the coefficients of the dummy variables of *dow_t_*. For temperature, the 2-day moving average (lag 0-1) was determined since the generalized cross-validation (GCV) score was the lowest among the various lag-applied models; *dfs* for temperature, relative humidity, and time trend were determined based on the consensus model methodology of a recent large-scale multi-city time series study [4]. For the *df* of the long-term trend, we also constructed GAMs applying 1-14 *dfs* per year. The GCV score was the lowest when 7 *df* was applied in the all-cause mortality models (Supplementary Material 2).

The coefficients (*β_PM 2.5_*) obtained from the 7 cities were pooled with the same exposure lag structure using random-effects meta-analyses, and the mortality risk percentage change per 10 μg/m^3^ increase in PM_2.5_ concentration was estimated using the following equation:


(2) $$Percentage\;change\;(△\%)\;=\;(e^{pooled\;\beta *10}-1)\times 100$$

For the second step, when the average 24-hour mean PM_2.5_ concentration was ≥ 35 μg/m^3^, it was classified as a high-concentration day. To investigate whether the effect size of PM_2.5_ changed during prolonged exposure to a high PM_2.5_ concentration, we added a 6-stratum consecutive day variable (no high-concentration days, the first to fourth day, and the fifth day and beyond; a coding example is shown in Supplementary Material 3) and its effect modification term to PM_2.5_ concentration in the quasi-Poisson GAM was as follows:


(3) $$\log[E(Y_{t,c})]=\beta_{0}\;+\;\sum_{k=1}^{6}\beta_{k,\;l,\;c}\;*\;PM_{2.5\;t,\;l,\;c}\;*\;con\_day_{t,\;l,\;c,\;k}\;+\;s(temp_{t,c},\;df\;=\;6)\;+s(rh_{t,c},\;df\;=\;3)\;+s(time_{t},\;df\;=\;7\;*\;number\;of\;years)\;+\;\eta \;*\;factor\;(dow_{t})$$

Where *con_day_t, l, c, k_* are 6 strata dummy variables corresponding to whether day *t* is not a high-concentration day (*k*=1), the first (*k*=2) to fourth (*k*=5) high consecutive days, and the fifth day or more (*k*=6) applied lagged exposure (*l*) in city *c*, respectively. *β*_1, *l, c*_ to *β*_6, *l, c*_ are the effects of PM_2.5_ in the corresponding high consecutive day strata. The rest of the variables are the same as those in the basic GAM. These models allow a different mortality effect within the predefined consecutive day strata [18,19]. We excluded the durational effect itself from the models since we did not observe a difference in model fitness based on whether an independent effect was applied. Thereafter, we pooled the PM_2.5_ coefficients paired for each lag structure and level of consecutive day variables using random-effects meta-analyses and estimated the mortality risk percentage change using the process described above.

To investigate the possible effect modification by age group, the above analyses were performed for 2 stratified age groups: 20-64 years old and ≥ 65 years old. For sensitivity analyses, we designed 2-pollutant models with daily ozone and nitrogen dioxide concentrations to measure the robustness of the effects of PM_2.5_ and their patterns within a prolonged duration of exposure to a high concentration of PM_2.5_. As a final step, in order to examine whether the effect modification of consecutive high-concentration days on mortality was caused by different background concentrations on each consecutive day, we also constructed an effect modification model in the same manner as described above based on a 10 μg/m^3^ unit daily background concentration interval (< 10 to ≥ 60 μg/m^3^) and compared the effect size of PM_2.5_ in the 2 models.

All dataset setups and statistical analyses were performed using R version 3.6.3 (Foundation for Statistical Computing, Vienna, Austria). We used the “mgcv” package for GAM modeling and the “metafor” package for random-effects meta-analyses in R software. The statistical significance level for the 2-tailed tests was set at 0.05.

### Ethics statement

The Institutional Review Board of Dankook University, Korea, exempted this study from review since it exclusively used anonymous secondary data (IRB No. DKU 2021-03-042).

## RESULTS

Table 1 summarizes the non-traumatic all-cause, respiratory, and cardiovascular mortality rates in 7 major cities from 2006 to 2019. In the study area, the total numbers of non-traumatic all-cause, respiratory, and cardiovascular deaths were 1,314,829 (daily mean, 257.2), 120,094 (daily mean, 23.5), and 326,034 (daily mean, 63.8), respectively.

**Figure 1.**
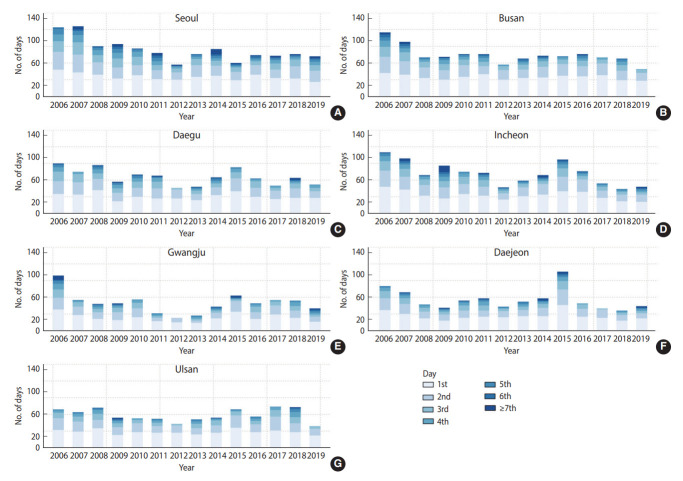
Distribution of consecutive days with high concentrations (35 μg/m^3^ or more) of fine particulate matter (PM_2.5_) in seven cities (A: Seoul, B: Busan, C: Daegu, D: Incheon; E: Gwangju, F: Daejeon, and G: Ulsan) in Korea by year from 2006 to 2019. The x-axis represents the year, and the y-axis represents the number of days with a high PM_2.5_ concentration (reference: 35 μg/m^3^) by year. A darker shade of blue indicates a greater number of days of exposure to high concentrations of PM_2.5_.

The mean concentration of PM_2.5_ throughout the study period ranged from 23.0 μg/m^3^ to 27.4 μg/m^3^, and the proportion of high PM_2.5_ concentration (an average 24-hour mean PM_2.5_ concentration of ≥ 35 μg/m^3^) in all cities during the study period ranged from 13.6% to 22.9%. Five or more consecutive high-concentration days accounted for 0.8-2.7% (Table 2). A declining trend was observed over the past 14 years. However, the annual average concentration in all 7 cities exceeded 20 μg/m^3^ in 2019, surpassing the annual governmental air quality guideline of 15 μg/m^3^ (Supplementary Material 4). Although the number of high-concentration days decreased every year up to 2019 (Supplementary Material 5), prolonged episodes of high concentrations did not disappear, and there were still consecutive days lasting more than a week in Seoul, Incheon, Gwangju, and Daejeon in 2019 (Figure 1).

The percentages of risk changes per 10 μg/m^3^ increase in PM_2.5_ concentration for all-cause, respiratory, and cardiovascular mortality in the lag 0-1 models were 0.33% (95% confidence interval [CI], 0.16 to 0.50), 0.47% (95% CI, -0.09 to 1.04), and 0.26% (95% CI, -0.08 to 0.60), respectively. The largest effects per 10 μg/m^3^ increase in all-cause, respiratory, and cardiovascular mortality were 0.33% (95% CI, 0.18 to 0.47, lag 0 model; more precise than lag 0-1), 0.62% (95% CI, -0.01 to 1.26, lag 0-2 model), and 0.39% (95% CI, 0.10 to 0.68, lag 0 model), respectively (Table 3).

In the models that applied the effect modification of consecutive high-concentration days, applying lag 0-1 exposure, we found increases in non-accidental all-cause mortality increases per 10 μg/m^3^ of 0.63% (95% CI, 0.20 to 1.06) on the first day and 0.36% (95% CI, 0.01 to 0.70) on the fourth day, which were higher than the effect size we found in the basic model. This pattern was similar for respiratory and cardiovascular mortality (Table 4). Considering the lag structures, we found higher effect sizes and clearer modified patterns in the lag 0-1 model (Supplementary Material 6).

In sensitivity analyses, the PM_2.5_ mortality effects were attenuated when we analyzed the 2-pollutant models that included daily ozone and nitrogen dioxide concentration. Nevertheless, the effect modification patterns of consecutive high-concentration days persisted (Supplementary Material 7).

**Figure 2.**
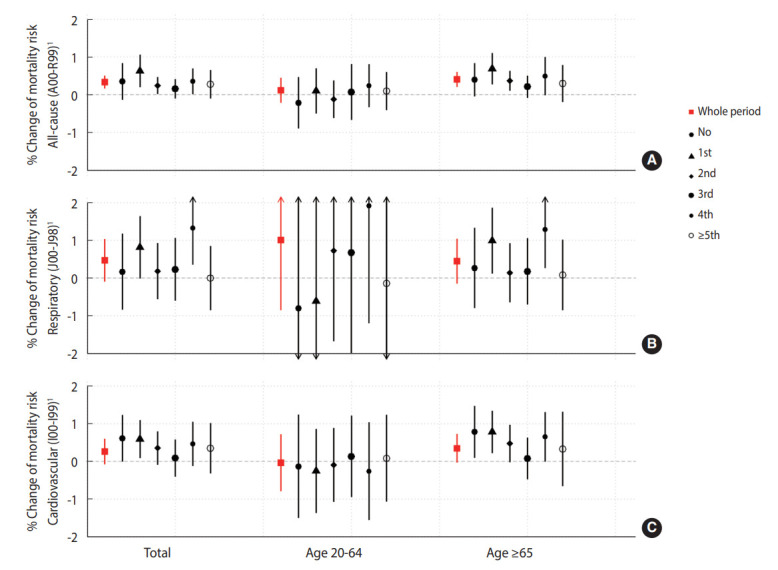
Non-traumatic all-cause (A), respiratory (B), and cardiovascular (C) mortality risk percent changes per 10 μg/m^3^ increase in the daily average fine particulate matter concentration and their modification by consecutive days of high concentration (35 μg/m^3^ or more) in a model with applied lag 0-1 exposure. This graph shows the percent of risk among the total population (left column), those aged 20-65 years (middle column), and those aged 65 years or older (right column). The red square boxes are estimates of the effects over the whole study period in basic models without applying consecutive day variables. Others represent estimates of six coefficients in the strata of consecutive days (no high-concentration days, the first to fourth days, and the fifth day and beyond) in the effect modification models. Vertical lines with point estimates indicate 95% confidence intervals. ^1^According to International Classification of Diseases, 10th edition code.

Although not statistically significant, we observed patterns of an increased effect on the fourth consecutive day in the 20-64 years age group for all-cause and respiratory mortality (Figure 2, Supplementary Material 8). The effect modification pattern was more pronounced in the ≥ 65 years age group (Figure 2, Supplementary Material 9).

In the sensitivity analysis with the effect modification according to the background concentration of PM_2.5_, we found that the effect sizes in all background concentration intervals were smaller than the effects of PM_2.5_ on the first and fourth consecutive days for all-cause mortality in the lag 0-1 model (Supplementary Material 10).

## DISCUSSION

We found a short-term positive effect of PM_2.5_ on mortality in this multi-city time-series study of 7 major Korean cities from 2006 to 2019. With continuous exposure to prolonged high concentrations of PM_2.5_ exceeding the daily mean of 35 μg/m^3^, the effects of PM_2.5_ on daily all-cause, respiratory, and cardiovascular mortality were higher on the first and fourth consecutive high-concentration days. In addition, this effect was mainly observed among the elderly (aged 65 years or older).

There have been several studies from Korea on the short-term effects of PM_2.5_ on mortality compared to the long-term effects; however, the effect sizes vary greatly depending on the spatiotemporal background of each study, the applied statistical model, and the exposure assessment [20]. Moreover, as of this study, only 2 multi-city designs have been published on the effect of particulate matter with a diameter of 10 microns or less (PM_10_) on mortality [4,21].

Two previous studies reported the short-term effects of PM_2.5_ on daily mortality for each city. Jung et al. [22] found that the total daily mortality risk of individuals aged ≥ 60 years increased by 0.36% per 10 μg/m^3^ increase of PM_2.5_ from 2000 to 2012 in Seoul. Kim et al. [23] found that the total daily mortality risk increased with a 10 μg/m^3^ increase of PM_2.5_ by 0.34%, 1.18%, and 0.43% in Seoul, Busan, and Incheon, respectively, from 2006 to 2012. We observed somewhat different effect sizes when compared to previous estimates. There were differences in the study period, which we expanded to 2019. The number of media reports in Korea of issues related to PM_2.5_ exposure has increased rapidly since 2012 [24], which was reflected in the increased rate of the everyday use of face masks due to improved risk awareness [25].

Two previous studies focused on the short-term effects of prolonged continuous exposure to a high concentration of PM. The first study observed an increased risk of cardiovascular and respiratory mortality after continuous exposure to high concentrations of PM in Beijing, China. Using GAM, the PM_2.5_ concentration variable was not added, and the categorical variable instead specified the duration of exposure to the high concentration of PM_2.5_ based on daily averages of 75 μg/m^3^, 85 μg/m^3^, 105 μg/m^3^, and 115 μg/m^3^, which were applied to the models. On the ninth consecutive day of exposure to a high concentration of PM_2.5_ of 105 μg/m^3^ or more, a 53% increase in the risk of cardiovascular mortality was reported among outdoor workers [12].

The second study observed a short-term durational effect of prolonged exposure to high concentrations of PM_10_ in 28 cities in China, Japan, and Korea. Using quasi-Poisson GAM, the effects of PM_10_ concentration and duration (number of consecutive days of exposure to 75 μg/m^3^ or more PM_10_) were separated. In Korea, the estimated increases in risk for each additional consecutive day of exposure to a high concentration of PM_10_ were 0.48% for non-traumatic all-cause mortality, 0.48% for cardiovascular mortality, and 1.13% for respiratory mortality [13].

Based on these 2 previous studies, we thought that it would be necessary to investigate whether the short-term mortality effect of PM_2.5_ was modified to a greater degree when exposure to a high concentration of PM persisted. To the best of our knowledge, this is the first study to report the effect modification of the short-term effect of PM_2.5_ after continued exposure to high concentrations.

A novel finding of this study was that the short-term effects of PM_2.5_ on daily mortality on the first and fourth consecutive days of exposure to high concentrations of PM_2.5_ were greater than the estimated effect for the entire period. From a short-term perspective, it was notable that the risk did not increase linearly as continuous exposure to high concentrations of PM_2.5_ increased; instead, the risk increased starting at the first transition to a day of high concentrations and at a later point, such as, for example, the fourth consecutive day of exposure.

We did not observe any reported biological mechanism related to effect modification. However, mortality displacement has been observed in previous studies on short-term air pollution epidemiology. This means that air pollution can cause mortality in frail individuals several days or weeks sooner than if they had not been exposed to air pollution. Thus, the mortality effect would be lower than expected after an initial risk increase [26,27]. In fact, we found that the effect on the first consecutive day of exposure to a high concentration of PM_2.5_ was remarkable among those aged ≥ 65 years. Among those aged 20-64 years, we did not observe a significant change in the effect due to the relatively low number of deaths. Nevertheless, even in the less vulnerable group, the point estimates were the greatest on the fourth day for all-cause and respiratory mortality. We speculate that another mortality displacement due to exposure to high concentrations of PM_2.5_ was revealed in this study. Furthermore, when high concentrations persisted for several days, relatively less frail individuals could also be affected by air pollution.

There is still insufficient understanding of intermittent episodes of high concentrations of PM_2.5_ in Korea, as well as spatial differences in the contribution sources and components of PM_2.5_ [11]. Increases in sulfate, nitrate, and ammonium ion concentrations have recently been more pronounced than organic carbon, inorganic carbon, and heavy metals in most consecutive high-concentration episodes without yellow dust storms in the western, central, and southeastern parts of the Korean Peninsula [11,28].

Despite the findings reported in Korea, evidence of the effects of sulfate, nitrate, and ammonia ions is still insufficient and inconsistent [29-32]. However, a recent meta-analysis found a statistically significant short-term increase in the risk of cardiovascular mortality due to nitrate and sulfate exposure and respiratory hospitalization due to nitrate exposure [33]. The effect of PM_2.5_ on daily mortality may increase with consecutive days of exposure to high concentrations of PM_2.5_ due to variations in the PM composition, particularly increases in nitrates and sulfates.

In the sensitivity analysis, we estimated the relative risks for all-cause mortality across 10 μg/m^3^ intervals of background PM_2.5_ concentration. A recent study in China showed that the relative risks of total and cardiovascular mortality increased as the background concentration increased [34]. We observed higher effect estimates on the first and fourth consecutive days than those derived from high PM_2.5_ concentration intervals of > 40 μg/m^3^ (Supplementary Material 10); therefore, the effect modification we observed was likely not due to the background concentration effect on the duration of exposure to high concentrations of PM_2.5_.

This study has several limitations. First, there may have been misclassifications in exposure assessment. In other words, local variations in the PM_2.5_ concentration within a single metropolitan area could not be reflected. In addition, the specific causes of regional and temporal variations in the effect of PM_2.5_ exposure could not be explained in our study. Future studies are needed to investigate the effect of applying the components of PM_2.5_ to the models as proposed in our study.

Despite these shortcomings, this study has several strengths. Among time series studies of the effect of PM_2.5_ exposure on short-term mortality in Korea, this study examined the longest time period at 14 years and was the first multi-city study of this type, including more than half of the entire Korean population. Moreover, by focusing on changes in the effect size of PM_2.5_ after persistent exposure to high concentrations, which is a challenge in East Asia, we found that the mortality effect of PM_2.5_ exposure may increase on a short-term basis even more during periods of exposure to high concentrations. Further epidemiological studies in other East Asian regions are needed.

We found a greater effect on daily mortality for the day when the duration of exposure to a high concentration of PM_2.5_ began and when exposure lasted for approximately 4 days. The elderly may be more affected by persistent exposure to high concentrations of PM_2.5_. Health authorities should encourage seniors or frail individuals to refrain from outdoor activity and wear a mask on days when there is a high concentration of PM_2.5_ that is expected to persist for several days. When the causes of episodes of high concentrations of PM_2.5_ and their components are identified, our results can be used as scientific evidence to support public risk communication and policy-making.

## Figures and Tables

**Figure f3-epih-44-e2022052:**
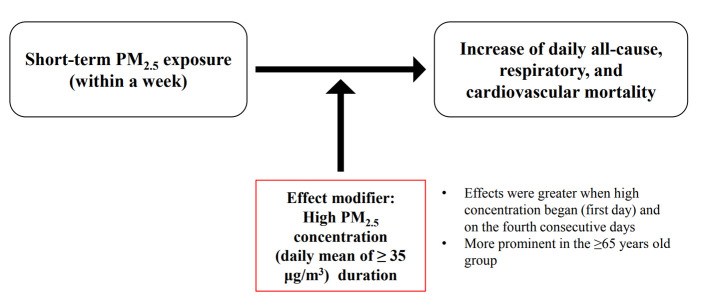


**Table 1.** All-cause (non-traumatic), respiratory, and cardiovascular mortality in 7 major cities in Korea from 2006 to 2019

City	Population in 2019	All-cause mortality (non-traumatic)		Respiratory mortality		Cardiovascular mortality	
		Total	Daily	Total	Daily	Total	Daily
Seoul	9,729,107	517,265	101.2±13.7	43,043	8.4±4.1	117,750	23.0±5.5
Busan	3,413,841	256,534	50.2±8.8	23,763	4.6±2.7	72,014	14.1±4.2
Daegu	2,438,031	157,211	30.7±6.6	15,373	3.0±2.1	42,042	8.2±3.0
Incheon	2,957,026	160,818	31.5±7.0	15,425	3.0±2.1	40,721	8.0±2.9
Gwangju	1,456,468	84,898	16.6±4.6	9,342	1.8±1.6	19,419	3.8±2.0
Daejeon	1,474,870	81,039	15.8±4.4	7,798	1.5±1.3	18,759	3.7±2.0
Ulsan	1,148,019	57,064	11.2±3.6	5,350	1.0±1.1	15,329	3.0±1.8
Total	22,617,362	1,314,829	257.2±31.5	120,094	23.5±9.8	326,034	63.8±10.4

Values are presented as number or mean±standard deviation.

**Table 2.** Daily fine particulate matter (PM_2.5_) concentrations and the proportion of exposure to high concentrations in seven major cities in Korea for 5,113 days from 2006 to 2019

City	Daily PM_2.5_ (μg/m^3^)	High-concentration days^1^	High-concentration duration^1^				
			1st day	2nd day	3rd day	4th day	5th day or more
Seoul	27.4±15.8	1,171 (22.9)	492 (9.6)	281 (5.5)	169 (3.3)	92 (1.8)	137 (2.7)
Busan	26.6±12.9	1,039 (20.3)	484 (9.5)	266 (5.2)	140 (2.7)	70 (1.4)	79 (1.5)
Daegu	25.3±13.2	919 (18.0)	426 (8.3)	238 (4.7)	132 (2.6)	65 (1.3)	57 (1.1)
Incheon	26.0±14.4	1,008 (19.7)	457 (8.9)	257 (5.0)	130 (2.5)	69 (1.3)	93 (1.8)
Gwangju	23.0±12.9	693 (13.6)	321 (6.3)	175 (3.4)	89 (1.7)	50 (1.0)	57 (1.1)
Daejeon	23.7±13.4	778 (15.2)	365 (7.1)	199 (3.9)	103 (2.0)	53 (1.0)	57 (1.1)
Ulsan	23.9±12.8	825 (16.1)	397 (7.8)	222 (4.3)	110 (2.2)	53 (1.0)	41 (0.8)

Values are presented as mean±standard deviation or number (%).

1 When the average 24-hour mean PM_2.5_ concentration was 35 μg/m^3^ or more, it was defined as a high-concentration day; We then specified the number of days of high concentrations for the duration.

**Table 3.** Mortality effects of short-term ambient fine particulate matter (PM_2.5_) exposure in seven major cities in Korea from 2006 to 2019

Lag structure^2^	All-cause (A00-R99)^1^			Respiratory (J00-J98)^1^			Cardiovascular (I00-I99)^1^		
	% Change^3^	95% CI		% Change^3^	95% CI		% Change^3^	95% CI	
		LL	UL		LL	UL		LL	UL
Single-day									
0	0.33	0.18	0.47	0.27	-0.21	0.77	0.39	0.10	0.68
1	0.16	0.02	0.31	0.42	-0.06	0.91	0.00	-0.29	0.29
2	-0.05	-0.20	0.09	0.37	-0.09	0.84	-0.33	-0.60	-0.05
3	-0.05	-0.19	0.09	-0.02	-0.64	0.61	-0.31	-0.58	-0.04
4	-0.20	-0.41	0.00	-0.02	-0.47	0.43	-0.30	-0.87	0.28
5	-0.33	-0.55	-0.11	-0.53	-0.97	-0.08	-0.25	-0.62	0.12
6	-0.29	-0.42	-0.15	-0.01	-0.45	0.44	-0.22	-0.49	0.05
Moving average									
0-1	0.33	0.16	0.50	0.47	-0.09	1.04	0.26	-0.08	0.60
0-2	0.25	0.05	0.44	0.62	-0.01	1.26	0.01	-0.37	0.40
0-3	0.19	-0.02	0.40	0.62	-0.07	1.32	-0.17	-0.59	0.25
0-4	0.06	-0.17	0.29	0.52	-0.26	1.31	-0.34	-0.96	0.28
0-5	-0.14	-0.43	0.16	0.24	-0.55	1.04	-0.47	-1.19	0.26
0-6	-0.28	-0.61	0.04	0.22	-0.62	1.07	-0.55	-1.24	0.15

City-specific estimated PM_2.5_ effects were from quasi-Poisson generalized additive models and pooled with the same exposure lag structure using random-effects meta-analyses. CI, confidence interval; LL, lower limit; UL, upper limit.

1 According to International Classification of Diseases, 10th edition code.

2 Single-day: the average 24-hour mean PM_2.5_ concentration from 0 (same day) to 6 days before; moving average: the average PM_2.5_ concentration over the current and previous day and the current to before the sixth day.

3 The % increase in mortality risk per 10 μg/m^3^ increase in PM_2.5_ concentration.

**Table 4.** Mortality effects of short-term ambient fine particulate matter (PM_2.5_) exposure and their modification by consecutive days of high PM_2.5_ concentration in seven major cities in Korea from 2006 to 2019

Variables	All-cause (A00-R99)^1^			Respiratory (J00-J98)^1^			Cardiovascular (I00-I99)^1^		
	% Change^2^	95% CI		% Change^2^	95% CI		% Change^2^	95% CI	
		LL	UL		LL	UL		LL	UL
Basic model	0.33	0.16	0.50	0.47	-0.09	1.04	0.26	-0.08	0.60
Effect modification model									
Consecutive days^3^									
No	0.35	-0.13	0.84	0.17	-0.84	1.18	0.61	0.00	1.23
1st day	0.63	0.20	1.06	0.82	-0.01	1.65	0.59	0.09	1.10
2nd day	0.24	0.02	0.46	0.18	-0.56	0.93	0.36	-0.09	0.80
3rd day	0.16	-0.10	0.41	0.23	-0.60	1.06	0.09	-0.40	0.58
4th day	0.36	0.01	0.70	1.33	0.36	2.31	0.47	-0.12	1.06
5th day or more	0.28	-0.10	0.65	-0.002	-0.85	0.85	0.35	-0.32	1.02

City-specific estimated PM_2.5_ effects were from quasi-Poisson generalized additive models with the 2-day moving average (the average over the current and previous day, lag 0-1) of PM_2.5_ concentration and pooled with the same consecutive-day strata using random-effects meta-analyses. CI, confidence interval; UL, upper limit; LL, lower limit.

1 According to International Classification of Diseases, 10th edition code.

2 The % increase in mortality risk per 10 μg/m^3^ increase in PM_2.5_ concentration.

3 The 6-strata categorical variable designating the number of consecutive days with daily mean PM_2.5_ concentrations of 35 μg/m^3^ or more.

## SUPPLEMENTARY MATERIALS

Supplementary Material 1. The map of seven major cities (Seoul, Busan, Daegu, Incheon, Gwangju, Daejeon, and Ulsan) in Korea.

Supplementary Material 2. Sensitivity analysis for the degree of freedom of time trend in generalized additive model for all-cause, respiratory, and cardiovascular mortality

Supplementary Material 3. An example of setting consecutive day variables in the effect modification models according to the daily mean concentration of PM_2.5_ (reference level=35 μg/m^3^).

Supplementary Material 4. Daily mean PM_2.5_ concentration in seven major cities in Korea from 2006 to 2019.

Supplementary Material 5. Days and proportion of the daily mean PM_2.5_ concentration of 35 μg/m^3^ or more in seven major cities in Korea from 2006 to 2019 (5,113 days).

Supplementary Material 6. Mortality effects of short-term ambient PM_2.5_ exposure and their modification by consecutive days of exposure to high concentrations of PM_2.5_ in seven major cities in Korea from 2006 to 2019.

Supplementary Material 7. Mortality effects of daily ambient PM_2.5_ exposure and their modification by consecutive days of exposure to high concentrations of PM_2.5_ in seven major cities in Korea from 2006 to 2019, with and without adjustment for daily ozone and nitrogen dioxide concentration.

Supplementary Material 8. Mortality effects of short-term ambient PM_2.5_ exposure and their modification by consecutive days of exposure to high concentrations of PM_2.5_ in seven major cities in Korea from 2006 to 2019 among those aged 20 and 64 years.

Supplementary Material 9. Mortality effects of short-term ambient PM_2.5_ exposure and their modification by consecutive days of exposure to high concentrations of PM_2.5_ in seven major cities in Korea from 2006 to 2019 among those aged 65 years or more.

Supplementary Material 10. Comparison of the effect modifications of pooled effect estimates (all-cause mortality risk percent changes per 10 μg/m^3^ increase of daily lag 0-1) of the average of same-day exposure to PM_2.5_ and PM_2.5_ exposure 1 day prior. Left is according to consecutive days, and right is to background mean concentration interval (μg/m^3^).
