# Supplementary material for: Effect modification of consecutive high concentration days on the association between fine particulate matter and mortality: a multi-city study in Korea
Source: Epidemiol Health. 2022 Jun 9;44:e2022052. doi: 10.4178/epih.e2022052 (PMC9754921; doi:10.4178/epih.e2022052)
Supplement: Supplementary Material 1. — The map of seven major cities (Seoul, Busan, Daegu, Incheon, Gwangju, Daejeon, and Ulsan) in Korea. [file epih-44-e2022052-suppl1.docx]

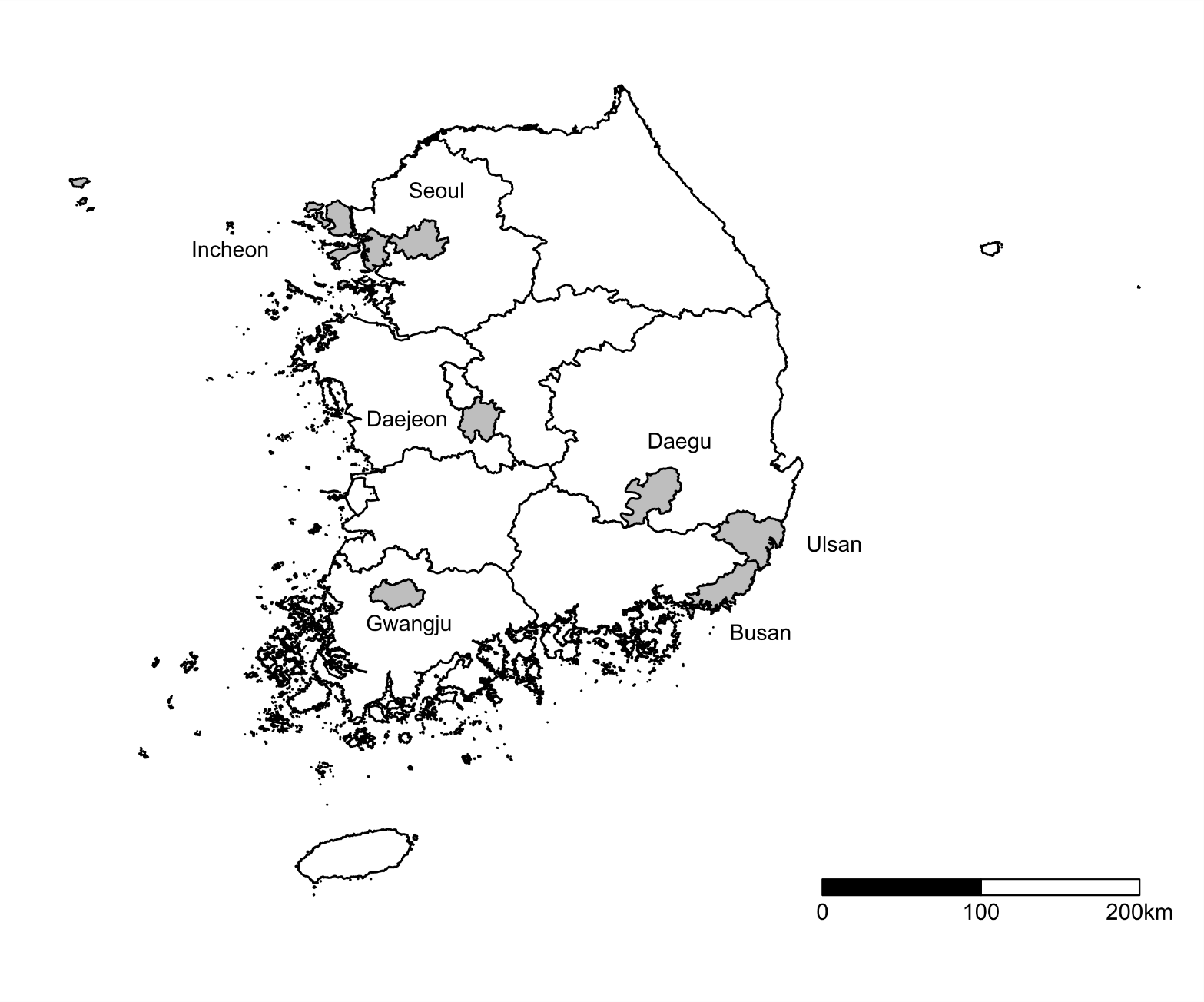


Supplementary Material 1. The map of seven major cities (Seoul, Busan, Daegu, Incheon, Gwangju, Daejeon, and Ulsan) in Korea.
