# Supplementary material for: Effect modification of consecutive high concentration days on the association between fine particulate matter and mortality: a multi-city study in Korea
Source: Epidemiol Health. 2022 Jun 9;44:e2022052. doi: 10.4178/epih.e2022052 (PMC9754921; doi:10.4178/epih.e2022052)
Supplement: Supplementary Material 2. — Sensitivity analysis for the degree of freedom of time trend in generalized additive model for all-cause, respiratory, and cardiovascular mortality [file epih-44-e2022052-suppl2.docx]

Supplementary Material 2. Sensitivity analysis for the degree of freedom of time trend in generalized additive model for all-cause, respiratory, and cardiovascular mortality

|  | All-cause (A00 - R99) | | | Respiratory (J00 - J98) | | | Cardiovascular (I00 - I99) | | |
| --- | --- | --- | --- | --- | --- | --- | --- | --- | --- |
| df | % change^1^ | 95% CIs | GCV^2^ | % change^1^ | 95% CIs | GCV^2^ | % change^1^ | 95% CIs | GCV^2^ |
| 1 | 0.99 | 0.69, 1.29 | 1.070 | 2.75 | 2.02, 3.49 | 1.153 | 0.95 | 0.45, 1.45 | 1.080 |
| 2 | 1.01 | 0.72, 1.29 | 1.063 | 2.93 | 2.14, 3.73 | 1.151 | 1.00 | 0.52, 1.48 | 1.080 |
| 3 | 0.38 | 0.17, 0.59 | 1.055 | 0.46 | -0.09, 1.02 | 1.137 | 0.25 | -0.08, 0.58 | 1.078 |
| 4 | 0.36 | 0.19, 0.53 | 1.053 | 0.49 | -0.07, 1.05 | 1.135 | 0.33 | -0.01, 0.66 | 1.080 |
| 5 | 0.30 | 0.13, 0.47 | 1.049 | 0.39 | -0.17, 0.95 | 1.135 | 0.27 | -0.07, 0.60 | 1.081 |
| 6 | 0.30 | 0.13, 0.48 | 1.048 | 0.48 | -0.09, 1.05 | 1.135 | 0.23 | -0.10, 0.57 | 1.083 |
| 7 | 0.33 | 0.16, 0.50 | 1.047 | 0.47 | -0.10, 1.04 | 1.135 | 0.26 | -0.08, 0.60 | 1.086 |
| 8 | 0.35 | 0.18, 0.52 | 1.049 | 0.52 | -0.05, 1.09 | 1.137 | 0.26 | -0.08, 0.60 | 1.089 |
| 9 | 0.36 | 0.19, 0.53 | 1.050 | 0.51 | -0.06, 1.09 | 1.139 | 0.30 | -0.04, 0.64 | 1.090 |
| 10 | 0.36 | 0.19, 0.54 | 1.050 | 0.43 | -0.14, 1.01 | 1.141 | 0.29 | -0.05, 0.64 | 1.093 |
| 11 | 0.35 | 0.18, 0.53 | 1.054 | 0.44 | -0.14, 1.02 | 1.144 | 0.29 | -0.06, 0.64 | 1.097 |
| 12 | 0.35 | 0.18, 0.53 | 1.055 | 0.48 | -0.10, 1.07 | 1.148 | 0.27 | -0.08, 0.62 | 1.099 |
| 13 | 0.35 | 0.18, 0.53 | 1.058 | 0.48 | -0.10, 1.07 | 1.150 | 0.30 | -0.05, 0.65 | 1.103 |
| 14 | 0.37 | 0.19, 0.54 | 1.061 | 0.53 | -0.06, 1.12 | 1.154 | 0.28 | -0.07, 0.64 | 1.105 |
| df: degree of freedom; CI: confidence interval; GCV: global cross validation City specific estimated effects were from quasi-Poisson generalized additive models (GAMs) with two-day moving average (the average over the current and previous day, lag 0-1) of PM_2.5_ concentration and they were pooled with the same dfs using random-effects meta analyses.  ^1^ The % increase mortality risks per an increase of 10 μg/m^3^ of PM_2.5_ concentration ^2^ mean value of GCVs from each of the 7 city GAMs with the same time trend df and outcome  Additional notes: We set the df of time trend to 7 based on the previous large-scale study (a priori) and our all-cause mortality models. However, the degree of freedoms of time trend which have the lowest GCV value in the model for respirtory and cardiovascular mortality were 4 and 3, respectively. Comparing the difference of the pooled estimates (PM_2.5_ effect) according to df from 1 to 14, we used meta-regression model with df as predictor variable (df=7 as reference level). In the meta-regression model for respiratory mortality, we found no difference pooled PM_2.5_ effect estimates when df was set to 4 (lowest GCV) compared to 7 (p-value = 0.800). In terms of cardiovascular mortality, we found no difference estimates when df was set to 3 (lowest GCV) compared to 7 (p-value = 0.987). | | | | | | | | | |
