# Supplementary material for: Effect modification of consecutive high concentration days on the association between fine particulate matter and mortality: a multi-city study in Korea
Source: Epidemiol Health. 2022 Jun 9;44:e2022052. doi: 10.4178/epih.e2022052 (PMC9754921; doi:10.4178/epih.e2022052)
Supplement: Supplementary Material 3. — An example of setting consecutive day variables in the effect modification models according to the daily mean concentration of PM2.5 (reference level=35 μg/m3). [file epih-44-e2022052-suppl3.docx]

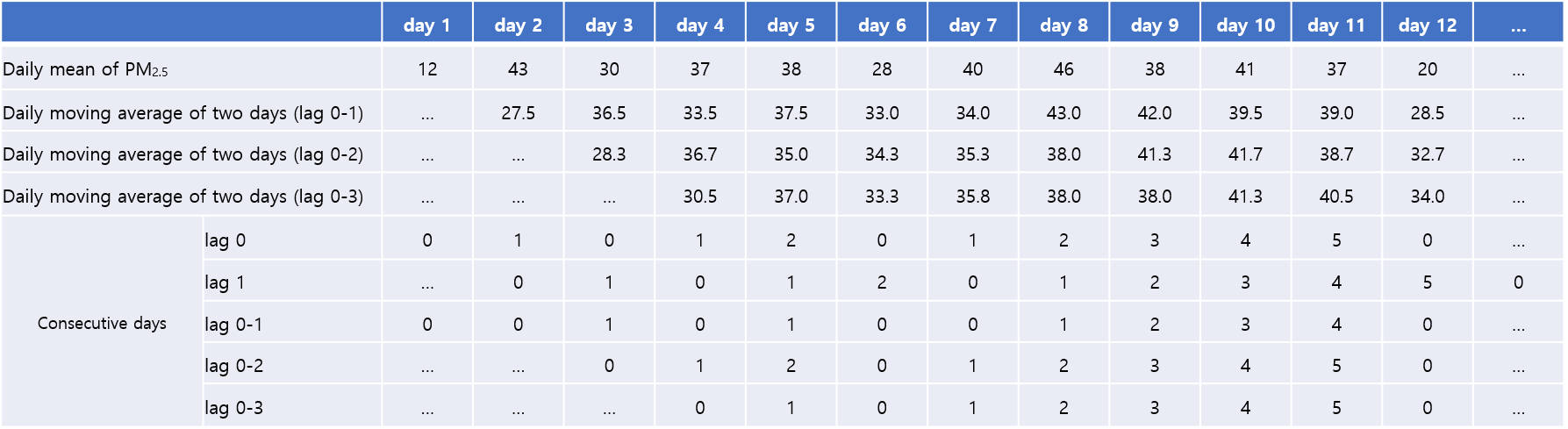


**Supplementary Material 3. An example of setting consecutive day variables in the effect modification models according to the daily mean concentration of PM_2.5_ (reference level=35 μg/m^3^).** We set lag for 24-hour mean PM_2.5_ exposure, 0 (the same day exposure) to 3 (exposure before 3 days) and 0-1 (the average of the same day and a day ago exposure) to 0-3 (the average of 4 days in the past including today) and designated consecutive days as the number of days in the high concentration duration. In a similar way, we also applied the lag structure for the consecutive days. Finally, we made consecutive day variable with six levels (No high day = 0, first to fourth day of high period = 1~4, fifth day or more of high period = 5) and applied our effect modification models.
