# Supplementary material for: Effect modification of consecutive high concentration days on the association between fine particulate matter and mortality: a multi-city study in Korea
Source: Epidemiol Health. 2022 Jun 9;44:e2022052. doi: 10.4178/epih.e2022052 (PMC9754921; doi:10.4178/epih.e2022052)
Supplement: Supplementary Material 4. — Daily mean PM2.5 concentration in seven major cities in Korea from 2006 to 2019. [file epih-44-e2022052-suppl4.docx]

Supplementary Material 4. Daily mean PM_2.5_ concentration in seven major cities in Korea from 2006 to 2019.

|  | Daily PM_2.5_ concentration [㎍/m^3^, mean (SD)] | | | | | | | | | | | | | | |
| --- | --- | --- | --- | --- | --- | --- | --- | --- | --- | --- | --- | --- | --- | --- | --- |
| City | Entire period | 2006 | 2007 | 2008 | 2009 | 2010 | 2011 | 2012 | 2013 | 2014 | 2015 | 2016 | 2017 | 2018 | 2019 |
| Seoul | 27.4 (15.8) | 30.9 (17.6) | 32.2 (18.9) | 29.6 (16.2) | 30.4 (17.3) | 28.3 (15.9) | 27.7 (17.6) | 24.2 (11.5) | 26.8 (13.9) | 27.2 (14.9) | 24.5 (12.3) | 26.5 (11.3) | 25.3 (14.2) | 24.5 (16.1) | 25.7 (18.3) |
| Busan | 26.6 (12.9) | 31.3 (16.9) | 29.9 (14.8) | 26.9 (13.4) | 27.1 (12.4) | 27.0 (13.5) | 27.6 (13.8) | 25.8 (9.7) | 26.6 (10.7) | 26.7 (11.5) | 25.6 (11.7) | 26.9 (12.5) | 25.6 (11.7) | 23.6 (12.4) | 21.9 (11.3) |
| Daegu | 25.3 (13.2) | 28.3 (16.2) | 27.1 (13.9) | 29.6 (16.4) | 25.1 (12.5) | 27.0 (15.1) | 25.4 (14.2) | 23.1 (9.6) | 24.8 (11.6) | 25.1 (12.5) | 25.8 (12.2) | 24.7 (10.6) | 23.2 (11.3) | 22.3 (12.6) | 22.8 (12.6) |
| Incheon | 26.0 (14.4) | 29.9 (16.2) | 30.1 (16.6) | 27.2 (14.1) | 28.5 (16.0) | 27.1 (15.1) | 26.8 (16.2) | 23.8 (10.8) | 24.8 (12.0) | 25.8 (12.8) | 28.2 (14.8) | 25.6 (11.4) | 23.6 (12.7) | 20.7 (13.0) | 22.4 (15.2) |
| Gwangju | 23.0 (12.9) | 27.7 (14.3) | 24.2 (13.6) | 25.0 (12.5) | 23.8 (12.1) | 22.5 (15.9) | 21.5 (13.9) | 19.4 (8.5) | 20.9 (10.6) | 21.8 (12.0) | 24.7 (13.4) | 22.9 (11.0) | 23.4 (11.4) | 22.6 (13.5) | 21.0 (14.4) |
| Daejeon | 23.7 (13.4) | 25.7 (15.8) | 24.9 (14.8) | 24.3 (12.1) | 23.4 (11.9) | 24.0 (13.4) | 25.4 (15.3) | 22.1 (10.7) | 23.8 (12.7) | 23.3 (13.3) | 28.1 (15.7) | 22.8 (11.5) | 21.5 (10.9) | 20.0 (11.6) | 22.0 (14.7) |
| Ulsan | 23.9 (12.8) | 25.3 (17.3) | 24.9 (14.1) | 26.1 (14.2) | 23.9 (11.9) | 23.9 (14.0) | 23.9 (13.7) | 23.0 (9.6) | 24.4 (10.9) | 23.9 (11.9) | 24.0 (11.9) | 23.4 (10.9) | 25.1 (12.2) | 22.9 (12.8) | 20.6 (11.4) |
