# Supplementary material for: Effect modification of consecutive high concentration days on the association between fine particulate matter and mortality: a multi-city study in Korea
Source: Epidemiol Health. 2022 Jun 9;44:e2022052. doi: 10.4178/epih.e2022052 (PMC9754921; doi:10.4178/epih.e2022052)
Supplement: Supplementary Material 5. — Days and proportion of the daily mean PM2.5 concentration of 35 μg/m3 or more in seven major cities in Korea from 2006 to 2019 (5,113 days). [file epih-44-e2022052-suppl5.docx]

Supplementary Material 5. Days and proportion of the daily mean PM_2.5_ concentration of 35 μg/m^3^ or more in seven major cities in Korea from 2006 to 2019 (5,113 days).

|  | Days (N, %) with 35 μg/m^3^ or more (daily mean PM_2.5_ concentration) | | | | | | | | | | | | | | | |
| --- | --- | --- | --- | --- | --- | --- | --- | --- | --- | --- | --- | --- | --- | --- | --- | --- |
| City | Entire period | 2006 | 2007 | 2008 | 2009 | 2010 | 2011 | 2012 | 2013 | 2014 | 2015 | 2016 | 2017 | 2018 | 2019 |  |
| Seoul | 1171 (22.9) | 124 (34.0) | 126 (34.5) | 90 (24.6) | 94 (25.8) | 86 (23.6) | 78 (21.4) | 57 (15.6) | 76 (20.8) | 85 (23.3) | 60 (16.4) | 74 (20.2) | 73 (20.0) | 76 (20.8) | 72 (19.7) |  |
| Busan | 1039 (20.3) | 115 (31.5) | 98 (26.8) | 70 (19.1) | 71 (19.5) | 76 (20.8) | 76 (20.8) | 57 (15.6) | 68 (18.6) | 73 (20.0) | 72 (19.7) | 76 (20.8) | 70 (19.2) | 68 (18.6) | 49 (13.4) |  |
| Daegu | 919 (18.0) | 90 (24.7) | 75 (20.5) | 87 (23.8) | 57 (15.6) | 70 (19.2) | 68 (18.6) | 46 (12.6) | 48 (13.2) | 66 (17.8) | 83 (22.7) | 63 (17.2) | 50 (13.7) | 64 (17.5) | 52 (14.2) |  |
| Incheon | 1008 (19.7) | 111 (30.1) | 99 (27.1) | 69 (18.9) | 86 (23.6) | 75 (20.5) | 73 (20.0) | 47 (12.8) | 59 (16.2) | 69 (18.9) | 97 (26.6) | 76 (20.8) | 55 (14.8) | 44 (12.1) | 48 (13.2) |  |
| Gwangju | 693 (13.6) | 99 (27.1) | 55 (15.1) | 48 (13.1) | 49 (13.4) | 57 (15.3) | 31 (8.5) | 23 (6.3) | 27 (7.4) | 43 (11.8) | 63 (17.3) | 49 (13.4) | 55 (15.1) | 54 (14.8) | 40 (11.0) |  |
| Daejeon | 778 (15.2) | 80 (21.9) | 69 (18.9) | 47 (12.8) | 41 (11.2) | 54 (14.8) | 58 (15.9) | 43 (11.7) | 52 (14.2) | 58 (15.9) | 106 (29.0) | 49 (13.4) | 41 (11.0) | 36 (9.9) | 44 (12.1) |  |
