# Supplementary material for: Effect modification of consecutive high concentration days on the association between fine particulate matter and mortality: a multi-city study in Korea
Source: Epidemiol Health. 2022 Jun 9;44:e2022052. doi: 10.4178/epih.e2022052 (PMC9754921; doi:10.4178/epih.e2022052)
Supplement: Supplementary Material 6. — Mortality effects of short-term ambient PM2.5 exposure and their modification by consecutive days of exposure to high concentrations of PM2.5 in seven major cities in Korea from 2006 to 2019. [file epih-44-e2022052-suppl6.docx]

Supplementary Material 6. Mortality effects of short-term ambient PM_2.5_ exposure and their modification by consecutive days of exposure to high concentrations of PM_2.5_ in seven major cities in Korea from 2006 to 2019.

|  |  | Percent change per 10 μg/m^3^ increase of PM_2.5_ (% change^1^, 95% CIs) | | | | | | |
| --- | --- | --- | --- | --- | --- | --- | --- | --- |
|  |  | Lag 0 | Lag 1 | Lag 2 | Lag 3 | Lag 0-1 | Lag 0-2 | Lag 0-3 |
| **All-cause (non-traumatic, A00 - R99) mortality** | | | | | | | | |
| Basic model | | 0.33 (0.18, 0.47) | 0.16 (0.02, 0.31) | -0.05 (-0.20, 0.09) | -0.05 (-0.19, 0.09) | 0.33 (0.16, 0.50) | 0.25 (0.05, 0.44) | 0.19 (-0.02, 0.40) |
| Effect modification model | |  |  |  |  |  |  |  |
| Consecutive days^2^ | No | 0.17 (-0.23, 0.58) | 0.31 (-0.15, 0.76) | -0.10 (-0.44, 0.25) | 0.06 (-0.21, 0.33) | 0.35 (-0.13, 0.84) | 0.37 (-0.21, 0.96) | 0.26 (-0.34, 0.87) |
|  | 1st days | 0.38 (0.06, 0.69) | 0.19 (-0.04, 0.43) | -0.12 (-0.31, 0.08) | 0.03 (-0.16, 0.22) | 0.63 (0.20, 1.06) | 0.42 (0.04, 0.80) | 0.27 (-0.06, 0.61) |
|  | 2nd days | 0.30 (0.10, 0.50) | 0.09 (-0.14, 0.33) | 0.00 (-0.30, 0.31) | 0.01 (-0.22, 0.25) | 0.24 (0.02, 0.46) | 0.32 (0.06, 0.58) | 0.48 (0.17, 0.79) |
|  | 3rd days | 0.12 (-0.19, 0.43) | 0.22 (-0.02, 0.45) | 0.03 (-0.28, 0.34) | -0.04 (-0.27, 0.18) | 0.16 (-0.10, 0.41) | 0.15 (-0.12, 0.41) | 0.09 (-0.23, 0.41) |
|  | 4th days | 0.36 (0.07, 0.65) | 0.23 (-0.06, 0.52) | -0.13 (-0.41, 0.16) | -0.11 (-0.40, 0.19) | 0.36 (0.01, 0.70) | 0.15 (-0.15, 0.45) | 0.17 (-0.19, 0.53) |
|  | 5th or more days | 0.32 (0.05, 0.58) | -0.03 (-0.30, 0.24) | -0.24 (-0.77, 0.29) | -0.30 (-0.73, 0.13) | 0.28 (-0.10, 0.65) | 0.04 (-0.22, 0.31) | -0.08 (-0.48, 0.32) |
| **Respiratory (J00 - J98) mortality** | | | | | | | | |
| Basic model | | 0.27 (-0.21, 0.77) | 0.42 (-0.06, 0.91) | 0.37 (-0.09, 0.84) | -0.02 (-0.64, 0.61) | 0.47 (-0.09, 1.04) | 0.62 (-0.01, 1.26) | 0.62 (-0.07, 1.32) |
| Effect modification model | |  |  |  |  |  |  |  |
| Consecutive days^2^ | No | -0.03 (-0.95, 0.90) | 0.41 (-0.51, 1.33) | 0.05 (-0.84, 0.96) | 0.33 (-0.92, 1.61) | 0.17 (-0.84, 1.18) | 0.42 (-0.65, 1.51) | 0.29 (-1.29, 1.90) |
|  | 1st days | 0.55 (-0.11, 1.21) | 0.05 (-0.61, 0.71) | 0.08 (-0.74, 0.90) | 0.07 (-0.80, 0.95) | 0.82 (-0.01, 1.65) | 0.77 (-0.20, 1.75) | 0.47 (-0.74, 1.70) |
|  | 2nd days | 0.10 (-0.61, 0.82) | 0.44 (-0.20, 1.09) | 0.50 (-0.12, 1.13) | 0.33 (-0.49, 1.15) | 0.18 (-0.56, 0.93) | 0.41 (-0.46, 1.29) | 0.57 (-0.43, 1.59) |
|  | 3rd days | 0.05 (-0.73, 0.84) | 0.85 (0.08, 1.63) | 0.79 (0.03, 1.55) | 0.43 (-0.33, 1.19) | 0.23 (-0.60, 1.06) | 0.63 (-0.24, 1.51) | 0.92 (-0.06, 1.91) |
|  | 4th days | -0.22 (-1.55, 1.13) | 1.40 (0.46, 2.34) | 0.35 (-0.58, 1.30) | -1.03 (-2.61, 0.57) | 1.33 (0.36, 2.31) | 1.30 (0.30, 2.31) | 1.12 (0.10, 2.15) |
|  | 5th or more days | 0.33 (-0.78, 1.46) | -0.09 (-1.03, 0.85) | -0.34 (-1.20, 0.52) | 0.12 (-0.72, 0.98) | -0.002 (-0.85, 0.85) | 0.17 (-0.68, 1.03) | 0.23 (-0.67, 1.14) |
| **Cardiovascular (I00 - I99) mortality** | | | | | | | | |
| Basic model | | 0.39 (0.10, 0.68) | 0.00 (-0.29, 0.29) | -0.33 (-0.60, -0.05) | -0.31 (-0.58, -0.04) | 0.26 (-0.08, 0.60) | 0.01 (-0.37, 0.40) | -0.17 (-0.59, 0.25) |
| Effect modification model | |  |  |  |  |  |  |  |
| Consecutive days^2^ | No | 0.59 (-0.19, 1.37) | 0.44 (-0.12, 1.01) | 0.19 (-0.39, 0.78) | -0.71 (-1.25, -0.17) | 0.61 (0.00, 1.23) | 0.17 (-0.48, 0.83) | 0.08 (-0.62, 0.78) |
|  | 1st days | 0.56 (0.16, 0.96) | 0.35 (-0.05, 0.74) | -0.31 (-0.70, 0.07) | -0.60 (-0.98, -0.22) | 0.59 (0.09, 1.10) | 0.33 (-0.25, 0.92) | 0.26 (-0.41, 0.93) |
|  | 2nd days | 0.60 (0.21, 0.98) | -0.25 (-0.70, 0.19) | -0.35 (-0.83, 0.14) | -0.25 (-0.62, 0.12) | 0.36 (-0.09, 0.80) | 0.20 (-0.32, 0.73) | 0.32 (-0.29, 0.93) |
|  | 3rd days | -0.05 (-0.52, 0.42) | -0.01 (-0.47, 0.45) | 0.04 (-0.41, 0.50) | -0.42 (-0.87, 0.02) | 0.09 (-0.40, 0.58) | 0.01 (-0.51, 0.53) | -0.34 (-0.93, 0.26) |
|  | 4th days | 0.32 (-0.25, 0.89) | 0.38 (-0.18, 0.95) | -0.13 (-0.69, 0.43) | -0.39 (-1.32, 0.55) | 0.47 (-0.12, 1.06) | -0.09 (-0.71, 0.54) | 0.00 (-0.61, 0.61) |
|  | 5th or more days | 0.46 (-0.41, 1.34) | 0.28 (-0.25, 0.81) | -0.12 (-0.68, 0.43) | -0.39 (-1.11, 0.33) | 0.35 (-0.32, 1.02) | -0.15 (-0.68, 0.37) | -0.38 (-1.03, 0.29) |
| City specific estimated PM_2.5_ effects were from quasi-Poisson generalized additive models (GAMs) and pooled with the same exposure lag structure and consecutive day strata using random-effects meta-analyses.  ^1^ The % increase mortality risks per 10 μg/m^3^ increase in PM_2.5_ concentration ^2^ the six-strata categorical variable designating the number of consecutive days with daily mean PM_2.5_ concentrations of equal or more than 35 μg/m^3^ | | | | | | | | |
