# Supplementary material for: Effect modification of consecutive high concentration days on the association between fine particulate matter and mortality: a multi-city study in Korea
Source: Epidemiol Health. 2022 Jun 9;44:e2022052. doi: 10.4178/epih.e2022052 (PMC9754921; doi:10.4178/epih.e2022052)
Supplement: Supplementary Material 8. — Mortality effects of short-term ambient PM2.5 exposure and their modification by consecutive days of exposure to high concentrations of PM2.5 in seven major cities in Korea from 2006 to 2019 among those aged 20 and 64 years. [file epih-44-e2022052-suppl8.docx]

Supplementary Material 8. Mortality effects of short-term ambient PM_2.5_ exposure and their modification by consecutive days of exposure to high concentrations of PM_2.5_ in seven major cities in Korea from 2006 to 2019 among those aged 20 and 64 years.

|  |  | Percent change per 10 ㎍/m^3^ increase of PM_2.5_ (% change^1^, 95% CIs) | | | | | | | |
| --- | --- | --- | --- | --- | --- | --- | --- | --- | --- |
|  |  | Lag 0 | Lag 1 | Lag 2 | Lag 3 | Lag 0-1 | Lag 0-2 | Lag 0-3 |  |
| **All-cause (non-traumatic, A00 - R99) mortality** | | | | | | | | | |
| Basic model | | 0.14 (-0.18, 0.46) | 0.06 (-0.22, 0.34) | 0.14 (-0.13, 0.41) | 0.11 (-0.15, 0.38) | 0.12 (-0.22, 0.45) | 0.18 (-0.19, 0.56) | 0.23 (-0.18, 0.64) |  |
| Effect modification model | |  |  |  |  |  |  |  |  |
| Consecutive days^2^ | No | -0.03 (-0.58, 0.52) | 0.09 (-0.65, 0.83) | -0.20 (-0.74, 0.34) | 0.24 (-0.30, 0.77) | -0.22 (-0.90, 0.46) | 0.22 (-0.62, 1.06) | 0.23 (-0.45, 0.92) |  |
|  | 1st days | 0.28 (-0.34, 0.91) | -0.25 (-0.64, 0.14) | 0.20 (-0.18, 0.58) | 0.11 (-0.26, 0.49) | 0.10 (-0.50, 0.70) | 0.02 (-0.69, 0.74) | -0.09 (-0.74, 0.57) |  |
|  | 2nd days | -0.15 (-0.53, 0.23) | 0.23 (-0.18, 0.66) | -0.04 (-0.52, 0.45) | -0.01 (-0.44, 0.42) | -0.12 (-0.62, 0.37) | 0.12 (-0.40, 0.64) | 0.40 (-0.20, 1.00) |  |
|  | 3rd days | 0.31 (-0.37, 1.00) | 0.04 (-0.57, 0.66) | 0.01 (-0.44, 0.46) | 0.12 (-0.32, 0.56) | 0.07 (-0.67, 0.81) | 0.15 (-0.35, 0.66) | 0.18 (-0.40, 0.76) |  |
|  | 4th days | 0.00 (-0.88, 0.90) | -0.01 (-0.63, 0.61) | -0.23 (-0.78, 0.32) | 0.42 (-0.12, 0.96) | 0.24 (-0.33, 0.81) | -0.04 (-1.08, 1.01) | 0.33 (-0.27, 0.93) |  |
|  | 5th or more days | 0.17 (-0.35, 0.69) | 0.09 (-0.42, 0.61) | 0.11 (-0.48, 0.70) | 0.29 (-0.34, 0.93) | 0.09 (-0.41, 0.60) | 0.08 (-0.43, 0.59) | 0.17 (-0.40, 0.74) |  |
| **Respiratory (J00 - J98) mortality** | | | | | | | | | |
| Basic model | | -0.07 (-1.68, 1.56) | 1.54 (-0.04, 3.14) | 0.24 (-2.10, 2.63) | 0.76 (-0.86, 2.40) | 1.01 (-0.85, 2.91) | 1.50 (-0.60, 3.63) | 1.26 (-1.71, 4.33) |  |
| Effect modification model | |  |  |  |  |  |  |  |  |
| Consecutive days^2^ | No | -1.35 (-4.92, 2.36) | 1.26 (-1.80, 4.41) | 2.43 (-0.60, 5.56) | 1.64 (-2.35, 5.80) | -0.80 (-4.09, 2.60) | -1.58 (-5.07, 2.02) | -0.51 (-4.25, 3.37) |  |
|  | 1st days | -0.53 (-2.70, 1.69) | 1.68 (-0.50, 3.89) | 0.78 (-2.51, 4.17) | 1.48 (-0.83, 3.84) | -0.61 (-3.32, 2.17) | -0.99 (-4.14, 2.26) | 0.56 (-3.05, 4.30) |  |
|  | 2nd days | 0.63 (-1.47, 2.77) | 0.82 (-1.29, 2.97) | 1.75 (-0.31, 3.85) | 0.16 (-2.88, 3.30) | 0.72 (-1.68, 3.18) | 1.39 (-2.01, 4.89) | 2.16 (-1.15, 5.58) |  |
|  | 3rd days | -0.77 (-3.34, 1.88) | 2.03 (-0.45, 4.58) | 0.89 (-3.17, 5.13) | 1.23 (-1.25, 3.79) | 0.67 (-1.99, 3.41) | 0.65 (-2.15, 3.54) | 0.32 (-3.44, 4.23) |  |
|  | 4th days | -0.30 (-3.38, 2.89) | 2.53 (-1.47, 6.70) | 1.14 (-1.95, 4.33) | 0.29 (-2.76, 3.44) | 1.92 (-1.20, 5.14) | 1.37 (-2.14, 5.01) | 1.39 (-1.91, 4.81) |  |
|  | 5th or more days | -0.28 (-3.19, 2.72) | -0.59 (-4.22, 3.17) | -1.42 (-4.54, 1.81) | 1.84 (-0.96, 4.71) | -0.14 (-2.95, 2.75) | -0.06 (-2.88, 2.83) | 0.88 (-2.61, 4.48) |  |
| **Cardiovascular (I00 - I99) mortality** | | | | | | | | | |
| Basic model | | 0.07 (-0.99, 1.15) | 0.06 (-0.58, 0.70) | 0.12 (-0.56, 0.80) | -0.36 (-0.96, 0.25) | -0.04 (-0.79, 0.72) | 0.05 (-0.79, 0.90) | -0.16 (-1.08, 0.77) |  |
| Effect modification model | |  |  |  |  |  |  |  |  |
| Consecutive days^2^ | No | 0.69 (-0.78, 2.19) | 0.58 (-0.67, 1.85) | 1.11 (-0.84, 3.11) | -0.18 (-1.39, 1.04) | -0.14 (-1.50, 1.25) | 0.06 (-1.40, 1.54) | 0.34 (-1.22, 1.93) |  |
|  | 1st days | 0.37 (-1.23, 2.00) | 0.10 (-0.79, 0.99) | 0.81 (-0.05, 1.68) | -0.59 (-1.44, 0.26) | -0.25 (-1.37, 0.87) | -0.22 (-1.52, 1.09) | 0.37 (-1.12, 1.88) |  |
|  | 2nd days | 0.09 (-0.76, 0.95) | 0.30 (-0.55, 1.16) | -0.06 (-1.38, 1.28) | -0.10 (-0.92, 0.72) | -0.10 (-1.07, 0.89) | 0.14 (-1.01, 1.30) | 0.28 (-1.07, 1.65) |  |
|  | 3rd days | 0.41 (-0.61, 1.44) | -0.37 (-1.73, 1.01) | 0.60 (-0.39, 1.61) | -0.83 (-1.83, 0.17) | 0.13 (-0.94, 1.22) | 0.34 (-0.80, 1.49) | -0.01 (-1.36, 1.36) |  |
|  | 4th days | -0.12 (-1.38, 1.15) | 0.15 (-1.52, 1.85) | -0.92 (-2.17, 0.35) | 0.56 (-0.67, 1.81) | -0.26 (-1.55, 1.04) | -0.25 (-1.58, 1.09) | -0.23 (-1.59, 1.14) |  |
|  | 5th or more days | 0.13 (-1.55, 1.85) | 0.28 (-0.89, 1.47) | 0.60 (-0.55, 1.76) | -0.10 (-1.25, 1.05) | 0.08 (-1.06, 1.24) | -0.18 (-1.34, 0.99) | -0.12 (-1.74, 1.52) |  |
| City specific estimated PM_2.5_ effects were from quasi-Poisson generalized additive models (GAMs) and pooled with the same exposure lag structure and consecutive day strata using random-effects meta-analyses.  ^1^ The % increase mortality risks per 10 μg/m^3^ increase in PM_2.5_ concentration ^2^ the six-strata categorical variable designating the number of consecutive days with daily mean PM_2.5_ concentrations of equal or more than 35 μg/m^3^ | | | | | | | | | |
