# Supplementary material for: Effect modification of consecutive high concentration days on the association between fine particulate matter and mortality: a multi-city study in Korea
Source: Epidemiol Health. 2022 Jun 9;44:e2022052. doi: 10.4178/epih.e2022052 (PMC9754921; doi:10.4178/epih.e2022052)
Supplement: Supplementary Material 10. — Comparison of the effect modifications of pooled effect estimates (all-cause mortality risk percent changes per 10 μg/m3 increase of daily lag 0-1) of the average of same-day exposure to PM2.5 and PM2.5 exposure 1 day prior. Left is according to consecutive days, and right is to background mean concentration interval (μg/m3). [file epih-44-e2022052-suppl10.docx]

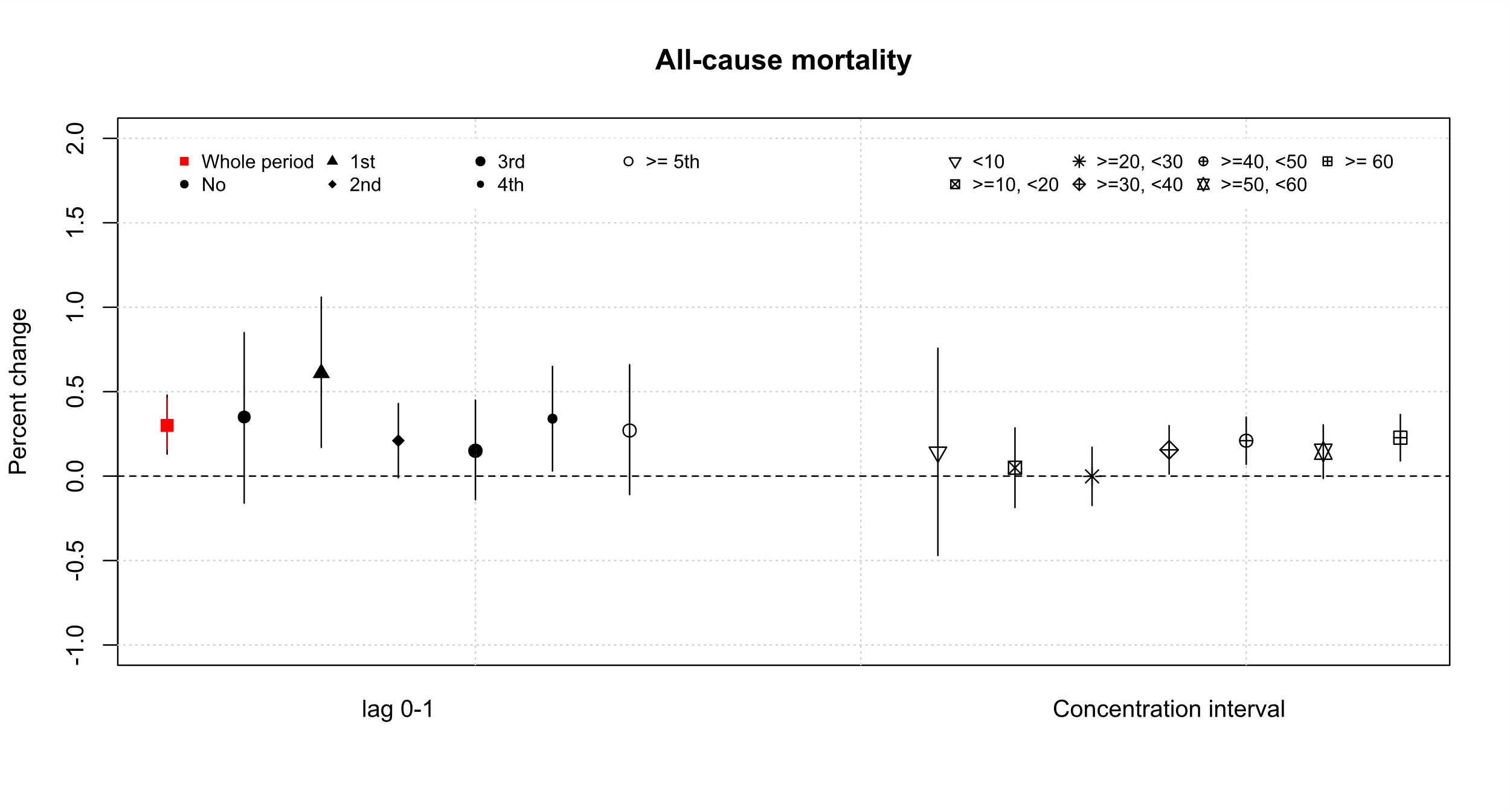


**Supplementary Material 10. Comparison of the effect modifications of pooled effect estimates (all-cause mortality risk percent changes per 10 μg/m^3^ increase of daily lag 0-1) of the average of same-day exposure to PM_2.5_ and PM_2.5_ exposure 1 day prior.** Left is according to consecutive days, and right is to background mean concentration interval (μg/m^3^).
